# Carbon nanotubes as neuroprotective agents

**DOI:** 10.37349/ent.2024.00071

**Published:** 2024-02-27

**Authors:** Daisy L. Wilson, Jyoti Ahlawat, Mahesh Narayan

**Affiliations:** Department of Chemistry and Biochemistry, the University of Texas at El Paso (UTEP), El Paso, Texas 79968, United States

**Keywords:** Carbon nanotubes, carbon nanomaterials, oxidative stress, amyloid fibrils, neurodegenerative disorders

## Abstract

Carbon nanotubes, an emerging class of carbon nanomaterials, possess tremendous potential for application in biotechnology and biomedicine particularly in neurological disorders. Carbon nanotubes owing to their fascinating properties have the potential to revolutionize medicine and technology, particularly in the realm of drug delivery, biosensing, bioimaging, and as therapeutic agents to tackle complex neurological disorders such as Alzheimer’s and Parkinson’s disease. In this review, a summary of the use of carbon nanotubes for neuropathological outcomes such as alleviating oxidative stress and amyloid formation, which are well-studied molecular outcomes associated with Alzheimer’s and Parkinson’s disease. In the end, challenges associated with the clinical testing of carbon nanotubes and possible ways to overcome them are highlighted.

## Introduction

**Figure 1.**
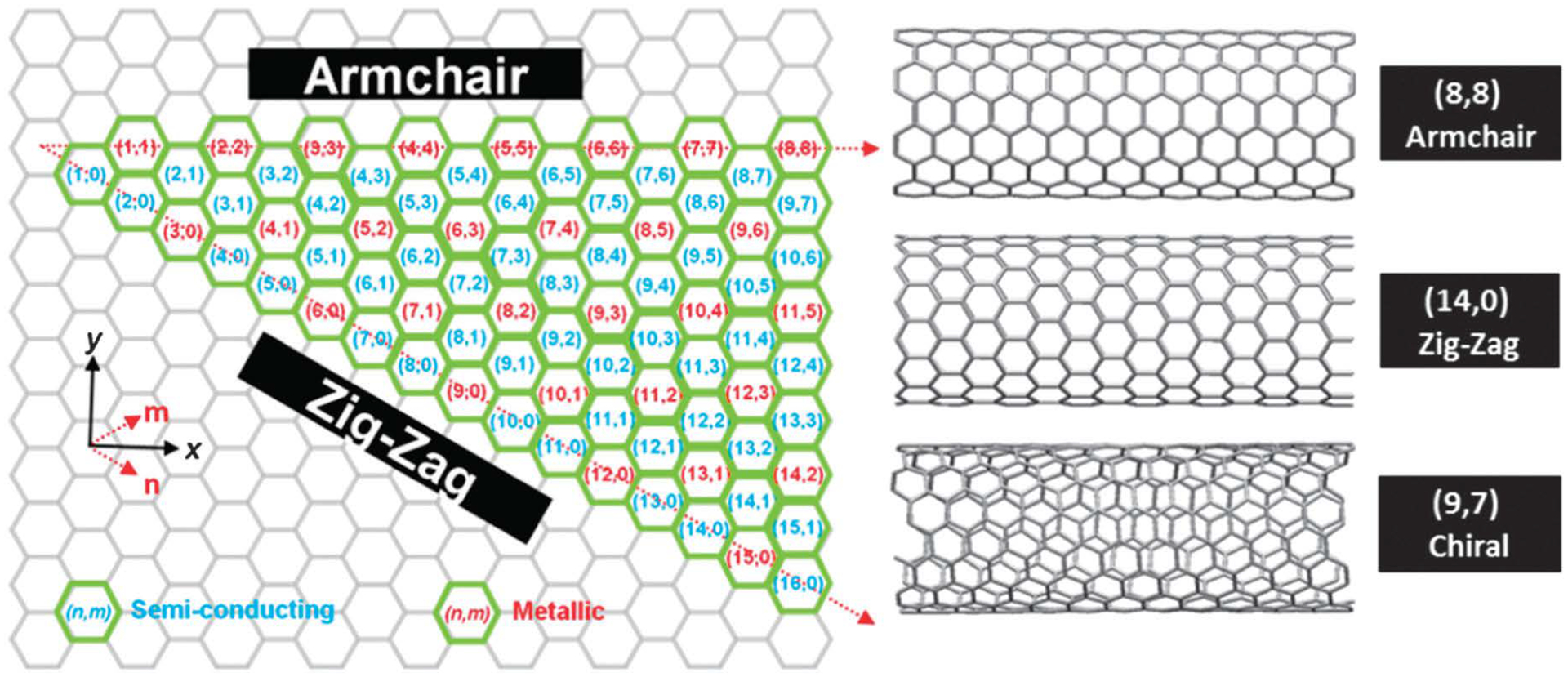
Helicity map of SWCNTs of different (*n*,*m*) chiral vectors *Note*. Reprinted from “Unweaving the rainbow: a review of the relationship between single-walled carbon nanotube molecular structures and their chemical reactivity” by Hodge SA, Bayazit MK, Coleman KS, Shaffer MS. Chem Soc Rev. 2012;41:4409–29 (https://pubs.rsc.org/en/content/articlelanding/2012/cs/c2cs15334c). © 2012 The Royal Society of Chemistry.

Carbon nanotubes (CNTs) are quasi-one-dimensional objects composed entirely of carbon and can be imagined as a single sheet of graphite rolled in a hollow cylindrical configuration with a diameter in the nanometer scale (Figure 1) [1–4]. These carbon nanomaterials (CNMs) can be classified into two main categories based on rolled-up graphene sheets as single-walled CNTs (SWCNTs, diameter range from 0.4 to 2 nm) and multiwalled CNTs (MWCNTs, diameter range from 1.4 to 100 nm). These cylindrical structures can be synthesized via arc discharge (electric discharge), chemical vapor deposition [metal-catalyzed decomposition of carbon source (methane, carbon monoxide, or acetylene) on a solid support such as aluminum oxide], and laser ablation (high-intensity laser pulses in a high-temperature inert atmosphere) methods [1–4]. Nevertheless, the chemistry of these CNMs is not that exciting as they are significantly unreactive. However, there are specific points in the structure that are more reactive than the others, e.g., defects (where carbon is missing) and curved-end caps (more strained as they deviate from the ideal planar geometry of graphite) [1–4]. These CNMs are insoluble in almost all organic solvents and aqueous solutions. Although dispersion in some solvents can be achieved by sonication, when the process is interrupted, immediate precipitation is observed [1–4]. However, the solubility issue can be overcome by chemical modifications resulting in enhanced integration of this nanomaterial into organic and biological systems [1–4]. CNTs exhibit interesting and exceptional chemical and physical properties such as high elasticity (exceeding 1 TPa), thermal conductivity (thermally stable up to 2,800°C in vacuum and can conductivity of 1,900 W m^−1^ K^−1^), ultra-lightweight small size with high surface area allowing multiple functionalization, high electrical conductivity (10^3^ S cm^−1^), unique electronic structures, and chemical inertness fueling research all around the world making them an ideal candidate for drug delivery and biosensing applications [5–10].

## Role of CNTs in neurodegenerative disorders

Neurodegeneration refers to the progressive loss of neurons which can be caused by many factors including aggregation and accumulation of misfolded amyloid proteins, inflammation, mitochondrial dysfunction, impaired autophagy-lysosomal activities, and oxidative stress to name a few [6–10]. It affects millions of people worldwide. Unfortunately, there remains an unmet diagnosis and treatment need for these disorders. Also, the development of drugs for these diseases is advancing at a very unsatisfying rate.

Over the past decades, great efforts have been made to exploit unusual and unique chemical and physical properties of CNTs for their use in biomedical applications including neurodegenerative disorders [6–10]. CNTs have shown great potential as tissue-engineered products (prosthetic neuronal devices), and as nanotools to accomplish tissue reconstruction and repair which are summarized in great detail elsewhere [6–10]. In this review, however, we summarize how CNTs can show neuroprotective effects by mitigating oxidative stress and dissolving and/or inhibiting the formation of amyloid fibrils.

### Impact of CNTs on amyloid fibrillation

Protein misfolding and aggregation begins with spontaneous self-assembly of polypeptides into a large, ordered, linear structure called amyloid fibrils. There are various intermediate states including nucleation (wherein no aggregates are formed but rather soluble mers interact with each other to form small nuclei that are later used as templates for further growth), oligomerization (wherein either soluble or insoluble oligomers are produced which leads to protofibrils and prefibrillar aggregates) and fibril formation in this process [5–7]. The amyloid fibril is composed of a cross-beta sheet in its core wherein beta-strands are stacked perpendicular to the long axis of the fibrils. Despite differences in the amyloid polypeptide precursor, resulting amyloid fibrils share this property and are involved in a number of human pathologies including Alzheimer’s disease (AD), Parkinson’s disease (PD), and Type II diabetes [5–7].

These amyloid aggregates can deposit inside various tissues and organs resulting in their damage and/or failure due to toxicity associated with their accumulation as such deposition prevents normal functioning of the affected tissue/organ/system. This phenomenon is referred to as amyloidosis. Despite intensive research and efforts geared toward deciphering the causes of amyloid misfolding and aggregation, there are no cures available to reverse this process. Therefore, the search for potent inhibitors has become an active research area for drug development against amyloidosis. Interestingly, over the years, CNTs have been explored for their potential to interact with amyloid proteins and affect the fibrillation process for therapeutic advantage [5–7].

In this section of the review article, we highlight and summarize articles that evaluate the inhibitory and disassembly properties of CNTs against amyloid fibrillation. We also describe various interactions between the two (CNM and amyloid protein) that result in these neuroprotective outcomes associated with CNTs and their derivatives.

Mo et al. [11] studied the inhibitory effect of hydroxylated CNTs on the aggregation of human insulin amyloid precursor protein (hIAPP) via computational and experimental studies. The authors studied the influence of SWCNTs on dimers which are the smallest oligomers formed during the early stage of protein aggregation. This process was studied both in the presence and absence of the SWCNTs. All-atom replica exchange molecular dynamics (REMD) simulations revealed that SWCNTs were able to bring changes in the secondary structure of the hIAPP dimers by reducing the beta-sheet content and by increasing the coil content of residues in regions of the dimers. The possible interactions preventing the hIAPP fibrillation were H-bonding, pi-stacking, and van der Waals interactions between SWCNTs and hIAPP dimers. These interactions could be weakening the inter-and intrapeptide interactions within and between dimers suggesting that SWCNTs can disassemble preformed amyloid fibrils [11].

The inhibitory effect (preventing amyloid fibril formation by interacting with intermediate stages) of SWCNTs functionalized with a hydroxy group (SWCNTs-OH) was explored experimentally using thioflavin T (ThT) assay, transmission electron microscopy (TEM), and atomic force microscopy (AFM). The obtained data revealed that SWCNTs were able to inhibit hIAPP misfolding by preventing self-assembly and hence aggregation of the amyloid protein. Thus, from the obtained results, it can be concluded that SWCNTs-OH can serve as a potential drug candidate to treat Type II diabetes [11].

Xie et al. [12] tested the effects of hydroxylated CNTs on amyloid beta (16–22) [Aβ (16–22)] peptides. To overcome the hydrophobic effect of pristine SWCNTs, the authors tested the effect of water-soluble SWCNTs on Aβ (16–22) aggregation. The molecular dynamics (MD) simulation data showed that hydroxylated SWCNTs were able to change the secondary structure from β-sheet rich to disordered coil aggregates. The driving force between this transition was found to be electrostatic interactions between the hydroxyl group of SWCNTs and positively charged amino acid on Aβ (16–22). Hydrophobic and pi-stacking were also involved in SWCNTs-peptide interactions and hence the inhibitory effect of Aβ (16–22) aggregation. The simulation data was supported by ThT and AFM imaging where both pristine and hydroxylated SWCNTs displayed an inhibitory effect on Aβ (16–22) fibrillization [12].

Li et al. [13] reported similar findings where the destabilization and inhibitory effect of SWCNTs on Aβ octamers (16–22) were studied. This work was performed in the presence and absence of SWCNTs using 110-ns all-atom REMD simulations. In the absence of SWCNTs, on average 44.5% beta-sheet content was recorded. Whereas, in the presence of SWCNTs, the same random Aβ (16–22) state shows a mostly disordered coil state. Also, a reduction in beta-sheet content to 7.9% was recorded. This inhibitory effect could be achieved by SWCNTs by slowing down the nucleation process. The main driving force behind such inhibitory effects were hydrophobic and pi-stacking interactions between the octamers and SWCNTs. Such interactions could prevent peptide-peptide interactions which are responsible for Aβ (16–22) aggregation. The study also showed that the introduction of SWCNTs destabilizes the pre-existing beta-sheet structures and promotes the formation of disordered coil aggregates and metastable beta-barrel-like structures. This result also gives hope that the fibrillation of Aβ (16–22) could be reversed if SWCNTs are added at the early stage of the aggregation process [13].

Song et al. [14] studied the effect of SWCNTs on amyloid-beta aggregate using all-atom MD of Aβ (1–42) trimers in the +/− of CNTs. This team previously reported the inhibitory effect of CNTs on β-sheet rich oligomer of Aβ (16–22). Here, in this work, the team studied the possible inhibitory interactions between the SWCNTs and Aβ (1–42) trimers. The possible interactions were hydrophobic and aromatic stacking between the SWCNTs and Aβ (1–42) trimers. The SWCNTs were able to distort the secondary structure of the peptide by hindering trimerization (by competing with Aβ-Aβ interactions), reducing alfa-helical content, and significantly promoting the formation of random coils. In conclusion, this study provides a mechanistic understanding of the interaction between SWCNTs and Aβ (1–42) peptide and the potential of SWCNTs to be developed into a therapeutic drug for AD [14].

Fu et al. [15] studied the effect of SWCNTs on Aβ (25–35) using atomic MD simulation. They observed the formation of β-barrel formation on the surface of SWCNTs. The analysis of the interaction between SWCNTs, water, and Aβ (25–35) oligomers shows that dehydration, Aβ-SWCNTs, and Aβ-Aβ interactions were the main driving force behind this. As observed in previous studies as well, intra-sheet H-bonding and hydrophobic interaction were the main driving forces behind β barrel formation. The results from this study are helpful in discovering the future prospect of using CNTs as a potential therapeutic to stop/halt/prevent Aβ fibrillation [15].

Zhao et al. [16] studied the effect the inhibitory effect of carboxylated SWCNTs on Aβ−40 fibrillation. The motivation behind this study was the need to develop inhibitors with the potential to inhibit Aβ aggregation, an approach that might prove effective and has the potential to alleviate and treat AD. The study involved both experimental and simulation work. In the experimental part, the authors observed the inhibitory and dissociative capability of COOH-SWCNTs via ThT and AFM images. The cytotoxicity studies performed using the 3-(4,5-dimethylthiazol-2-yl)-2,5-diphenyltetrazolium bromide (MTT) assay showed that COOH-SWCNTs were able to mitigate Aβ−40 induced toxicity *in vitro*. The MD studies, in line with previous findings, showed that COOH-SWCNTs destroyed the internal structural stability and β-sheet content via forming H-bonds, salt bridges, and pi-interactions with the Aβ−40 trimers. Consistent with previous findings, the present work also demonstrates the need to exploit the anti-Aβ potential of SWCNTs in the near future [16].

Wang et al. [17] also explored the effect of different functionalization on Aβ−42 fibrillation using MD simulation. The findings are in alignment with previous studies where H-bonding and Vander Waals interactions were shown to be the main driving force between Aβ-SWCNTs interaction. The additional finding from this study was that the presence of functional groups and charged amino acids on the surface of SWCNTs allowed electrostatic interaction between Aβ-SWCNTs. These interactions facilitated denaturation and destruction of Aβ−42 secondary structure and hence prevented aggregation. Consistent with previous findings, this work also demonstrates the excellent potential of functionalized SWCNTs as anti-AD treatment [17].

Lin et al. [18] explored the interplay between SWCNTs and pre-formed Aβ fibrils using AFM at a single SWCNT level. MD simulation, ThT, infrared spectroscopy (IR), and cytotoxicity studies were also performed alongside. The findings from this study were consistent with the previous one wherein a reduction in β-sheet content and partial destruction of pre-formed Aβ fibrils was recorded. Therefore, suppression and decomposition of Aβ fibrils by SWCNTs could be a promising strategy for developing therapy for AD [18].

**Figure 2.**
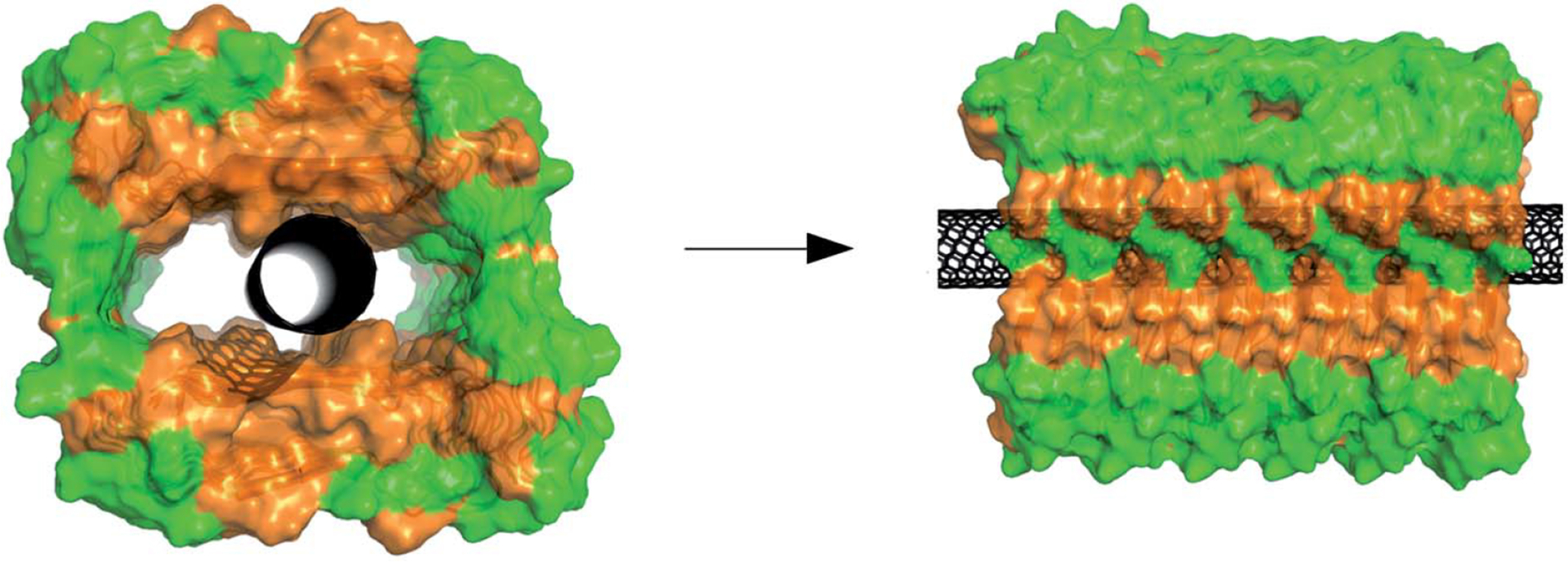
Illustration of a single walled nano tube (SWNT) located in the hollow core of an amyloid-beta peptide fibril. Orange and green represent hydrophobic and hydrophilic residues, and the black one is the CNT. Although this structure is theoretically possible, it is not backed by experimental evidence *Note*. Reprinted from “The Aβ peptide forms non-amyloid fibrils in the presence of carbon nanotubes” by Luo J, Wärmländer SK, Yu CH, Muhammad K, Gräslund A, Pieter Abrahams J. Nanoscale. 2014;6:6720–6 (https://pubs.rsc.org/en/content/articlelanding/2014/nr/c4nr00291a). © 2014 The Royal Society of Chemistry.

Luo et al. [19] investigated the effect of hydrophobic SWCNTs on the solubility, structure, and misfolding of Aβ peptides. This work is different from the previously summarized works as the authors report reduced cytotoxicity of SWCNTs in the presence of Aβ peptides. The authors also reported an influence of CNTs on Aβ fibrils nucleation and directing the formation of non-amyloid fibrils. The PH-dependent interaction between the amyloid and SWCNT was studied using ThT assay, nuclear magnetic resonance (NMR), circular dichroism (CD), and AFM imaging. This work demonstrates the possibility of the occurrence of non-amyloid fibrils, which could not be detected by ThT assay due to the working principle of dye [20]. ThT dye undergoes rotational immobilization around its central C-C bond (connecting benzothiazole and aniline rings) upon binding to the amyloid fibrils. Therefore, any fibrils not containing cross-beta sheet architecture cannot be detected by the ThT dye [20]. The results from ThT show that SWCNTs were able to inhibit the nucleation phase of the fibrillation. AFM data showed that Aβ helped in solubilizing hydrophobic SWCNTs by adsorbing them onto their surface. The NMR measurements showed an interaction between SWCNTs and Aβ peptide. This finding also shows that SWCNTs do not have an effect on the toxicity of Aβ oligomers, although Aβ did help in overcoming the toxicity aspect of SWCNTs (made it less toxic). Hence, it can be that proteins can also have protective effects on nanomaterials [19] (Figure 2).

### Potential role of CNTs in mitigating oxidative stress

Oxidative stress arises due to a serious imbalance in the redox state of a cell, which involves the generation of excess reactive oxygen species (ROS) and reactive nitrogen species (RNS) or dysfunction of radical detoxifying enzymes (superoxide dismutase, glutathione peroxidase, and catalase). This results in high levels of oxidized, unfunctional biomolecules such as proteins, DNA, RNA, carbohydrates, and lipids within the cells [21–22].

Oxidation of DNA can be very detrimental as it can affect the replication and transcription of crucial genes. Oxidation of DNA results in DNA strand breaks. For example, oxidation of nucleoside guanosine by hydroxyl radical generates 8-hydroxydeoxygyanosine (8-OH-dG) and 8-hydroxyguanosine (8OHD). 8-OH-dG is a major marker to detect DNA oxidation within the cells. Similarly, oxidation of RNA could result in nucleotide strand break and ribosomal dysfunction. On the other hand, the oxidation of lipids can be fatal too as it impacts the integrity of the cell membrane. The polyunsaturated fatty acids (PUFAs) in the cell membranes are most susceptible to oxidation. The PUFAs get peroxidized upon attack by hydroxy radicals resulting in isoprostane formation. Therefore, measuring the level of isoprostanes is a good marker to detect oxidative stress inside the cells. The other products formed during lipid peroxidation are 4-hydroxynonenal (HNE) and malondialdehyde (MDA). Both of them are aldehydes that have the potential to react with proteins and disrupt their function [21–22].

When ROS or RNS reacts with proteins, oxidative or nitrosative damage occurs either due to side chain oxidation or backbone fragmentation or misfolding which ultimately results in loss of proteins losing their biological function. Protein carbonylation involves the introduction of carbonyl groups in proteins via the oxidation of lysine, arginine, proline, and threonine residues. It can also be introduced by cleavage of peptide bonds via alfa-amidation or oxidation of glutamyl residues. Therefore, the measurement of protein carbonyls could be a good marker for estimating the extent of protein oxidation inside the cells. Oxidation can also result in the generation of advanced glycation products (AGEs), and other toxic species such as alcohols, aldehydes, and ketones. Other sources of ROS and RNS include astrocytes and microglia. ROS are also generated via Fenton chemistry wherein oxygen reacts with unregulated redox-active metals such as iron and copper.

This section highlights and summarizes research work testing the potential of CNTs in mitigating oxidative stress inside cells [21–22]. The free radical sponge property of CNTs arises from its sp^2^ hybridization which also allows them to be functionalized with various functional groups including biomolecules with antioxidant properties. It is important to evaluate this as oxidative stress is one of the major molecular outcomes associated with AD and PD. The other observation made during writing this section of the review article was that not much research on testing the antioxidant and free radical scavenging ability of CNTs for neurodegenerative disorders is out there. Therefore, highlighting the potential of using CNTs for this purpose was important.

Pegylated-SWCNTs were tested for their potential antioxidant effect on the nervous system [23]. The SWCNT-polyethylene glycol (PEG) was introduced into the rat brain and their effect on oxidative stress and morphology of the hippocampus was assessed. The results showed that at 0.5 mg/mL and 1 mg/mL, SWCNTs-PEG decreased total antioxidant capacity in the hippocampal region after one-day post-injection. However, SWCNTs-PEG at all tested concentrations were able to activate the antioxidant defense and alleviate ROS level after seven days post-injection. The authors hypothesized that the extended presence of SWCNTs-PEG in tissue can induce an antioxidant response. The authors also noted that SWCNTs-PEG had no effect on the contextual fear memory, locomotory activity, and spatial recognition memory of infused rats [23].

Gallic acid-functionalized MWCNT conjugate (GA-CNT) was synthesized as an acetylcholinesterase (AChE) inhibitor. Herein, GA-CNTs were proposed as an inhibitor of AChE and as an antioxidant [24]. To evaluate this, 2,2-diphenyl-1-picrylhydrazyl (DPPH), hydroxyl radical scavenging test, linoleic acid peroxidation inhibition, and total antioxidant activity, and AChE inhibition tests were performed. The authors also performed the hen’s egg test on the chorioallantoic membrane (HET-CAM) test to evaluate the biocompatibility of the synthesized material. The obtained results clearly demonstrate the potential of this nanoconjugate in the treatment of AD [24].

## Conclusions

Properties such as the ability to form pi-pi interactions with amyloid fibrils, large surface area to volume ratio for higher payload delivery, biocompatibility, and reduced toxicity upon surface functionalization enable CNTs as an ideal candidate to be used as drug against neurological diseases. In addition, its chemical stability, and unique electronic and mechanical properties have paved its ways in applications ranging from sensors to tissue supports to artificial muscles [5–10].

The biomedical application of CNTs is restricted due to their hydrophobic surface which makes them susceptible to aggregation in an aqueous environment. This can also result in their persistence in biological systems and unspecific bioaccumulation despite clearance mechanisms. However, surface functionalization with various biocompatible functional groups and biocompatible surfactants such as poly-oxyethylene sorbitan mono oleate 80 (PS80) can be performed to overcome this limitation. Functionalization can not only help with overcoming solubility-related aspects of CNTs but can also help reduce cytotoxicity and immunogenicity which is highly desirable and crucial for any biomedical application. Various methods that can be used to functionalize CNTs (covalently and non-covalently) have been summarized elsewhere [25]. The ability of CNTs to undergo functionalization makes them a great drug delivery candidate as they are capable of high payload delivery due to their high surface area. Functionalization of CNTs can also help in achieving targeted delivery of drugs [26–29]. They also possess thermal and optical properties which can be leveraged for photo-thermal and multimodal real-time tracking inside a biological system [30–31].

The other limitation associated with CNT delivery to the central nervous system is crossing the blood-brain barrier (BBB). As mentioned previously, surface functionalization and modifications could help but more research needs to be carried out *in vitro* and *in vivo* to exploit the great potential of CNTs [26–29]. Certain research work also shows that CNTs can elevate ROS and induce oxidative stress inside cells. Since organs like the spleen, kidney, and lungs are more vulnerable to oxidative stress compared to other organs of the body, more research on the antioxidant potential of CNTs inside biological systems needs to be performed before using them for medicinal properties [31–35].

Despite rapid development in synthesis and characterization methods of CNTs, relatively less is known about the interaction of CNTs with biological surfaces and long-term impact its exposure can have/bring in a biological environment. CNTs owing to their nanosize can access tissue or cells and offer significant potential in probing the mechanism of amyloid fibrilization, however little is known and understood whether such diagnosis and treatment by itself can cause any unintended toxicity. Therefore, the mechanism of action needs to be studied in detail to decipher the good and bad impact of CNTs inside a biological system before such therapies can be approved and/or implemented. Some of the questions that need to be addressed include: How are the CNTs showing their effect? Are they directly interacting with any surface receptor on cells and showing downstream effects? or if there is a receptor-mediated endocytosis? or some other form of cellular uptake of CNTs that results in the cellular effect that we see via cell-based or other assays? Such understanding of mechanism of action (MOA) will help in better functionalization and hence dose selection (pharmacokinetics) of the CNTs which is required to see therapeutic effect both *in vitro* and *in vivo* [26–29]. Also, it is important to produce chemically and structurally the same CNTs and overcome batch-to-batch variation that arises during the CNT synthesis process to observe consistent biological effects. Further research in this area is needed but the future of CNTs in neurological diseases looks promising.

## Abbreviations

AChE: acetylcholinesterase
AD: Alzheimer’s disease
AFM: atomic force microscopy
Aβ: amyloid beta
CNMs: carbon nanomaterials
CNTs: carbon nanotubes
hIAPP: human insulin amyloid precursor protein
MD: molecular dynamics
PEG: polyethylene glycol
RNS: reactive nitrogen species
ROS: oxygen species
SWCNTs: single-walled carbon nanotubes
ThT: thioflavin T
